# Corrosion resistance of straight and C-clasp stainless steel wrought wires in artificial saliva

**DOI:** 10.2340/biid.v13.46369

**Published:** 2026-07-02

**Authors:** Eri Hendra Jubhari, Baiq Zalfa Zahira Aurellia Nityasari, Rifaat Nurrahma

**Affiliations:** a Department of Prosthodontics, Faculty of Dentistry, Hasanuddin University, Makassar, Indonesia; b Hasanuddin University Dental Hospital, Makassar, Indonesia; c Faculty of Dentistry, Hasanuddin University, Makassar, Indonesia

**Keywords:** stainless steel, corrosion, saliva pH, wire bending, removable partial denture

## Abstract

**Background:**

Stainless steel wires are commonly used in removable partial dentures, but their corrosion resistance may be affected by intraoral pH changes and mechanical deformation, such as bending into C-clasps.

**Objective:**

To evaluate the corrosion resistance of straight and C-clasp stainless steel wires in artificial saliva (pH 5.5 and 7.0) and to assess the influence of bending on corrosion behavior.

**Materials and methods:**

An in vitro experimental study was conducted using four groups of stainless steel wires: straight pH 5.5, C-clasp pH 5.5, straight pH 7.0, and C-clasp pH 7.0. The samples were immersed for 4 weeks, with weekly weight measurements and corrosion rate calculations performed. Data were analyzed using repeated measures analysis of variance (ANOVA) or the Friedman test and two-way ANOVA for intergroup comparisons.

**Results:**

Two-way ANOVA revealed a significant effect of pH on corrosion behavior (*p* < 0.001), whereas wire configuration had no significant effect (*p* > 0.05). Acidic conditions (pH 5.5) resulted in significantly greater weight loss in both straight and C-clasp wires than neutral conditions, indicating increased corrosion in acidic environments. At pH 7.0, the corrosion rates were minimal or slightly negative, suggesting limited corrosion or minor weight changes.

**Conclusion:**

Environmental pH plays a central role in corrosion behavior, with acidic conditions accelerating degradation. No significant effect of bending into a C-clasp on corrosion susceptibility was observed under the present conditions. Maintaining an optimal oral pH may help preserve the integrity of stainless steel components in removable partial dentures.

**KEY MESSAGES**

1. Oral pH fluctuations significantly influence the corrosion rates of stainless steel wrought wire clasps, with acidic environments causing measurable changes in the degradation of the material.
2. Under the present experimental conditions, mechanical deformation (bending) did not significantly affect the corrosion behavior of stainless steel wires.
3. Understanding how pH variations and mechanical stress affect corrosion can help clinicians optimize clasp fabrication and anticipate the long-term performance of removable partial dentures.

## Introduction

### Background/Rationale

Tooth loss is a common oral condition that can impair mastication, speech, and esthetics, thereby affecting oral function and quality of life [1, 2]. Removable partial dentures are commonly used to replace missing teeth and restore their functions. One of the essential components of these prostheses is the clasp, which is typically fabricated from stainless steel wire and provides retention and stability during function [3–5]. Despite the favorable mechanical properties and corrosion resistance of stainless steel [6], metal components placed in the oral cavity may still undergo corrosion due to the complex oral environment [5, 7].

Corrosion is a degradation process caused by electrochemical reactions between a metal surface and its environment and several oral factors (including fluctuating pH, moisture, electrolytes, food substances, and microorganisms) can accelerate this process [6, 8, 9]. Stainless steel relies on a protective chromium oxide passive layer for corrosion resistance, similar to other base metal alloys [10]. This passive film is typically 2–5 nm thick and primarily composed of chromium oxide (Cr₂O₃), supported by a subsurface transition layer and a bulk metallic core containing Fe, Cr, Ni, and Mo [11–13]. When exposed to acidic media, this passive layer becomes more vulnerable to degradation, allowing aggressive ions such as Cl^–^ and H^+^ to penetrate the surface and promote the electrochemical dissolution of the underlying metal [12–14]. This passive film may also be compromised by clinical and laboratory procedures involved in clasp fabrication, such as repeated bending or improper manipulation with pliers [15]. Damage or rupture of the passive film exposes the underlying metal surface, facilitating oxidation, ion release, and initiation of localized corrosion mechanisms, such as pitting [16, 17].

Wrought stainless steel wire clasps are frequently chosen for their flexibility, minimal tooth coverage, ease of adjustment, and esthetic advantages [4]. Unlike cast clasps, wrought wire components undergo significant mechanical manipulation during fabrication, increasing the likelihood of structural alterations and passive layer disruption [4, 15]. Furthermore, variations in oral pH, which normally range from 6 to 7 [18] but may decrease to below 5.5 during carbohydrate fermentation [5] or as low as 2.5 under inflammatory conditions [19], can markedly accelerate corrosion rates and increase the release of metal ions, such as chromium and nickel [20]. Local and systemic effects of these ions have been reported, including mucosal discoloration, lichenoid lesions, burning sensations, hypersensitivity reactions, and potential oxidative or immunological impacts [20].

While previous studies have examined the corrosion behavior of various dental alloys, they often focus on basic forms such as flat sheets or straight wires, overlooking components that undergo significant mechanical deformation during fabrication [8, 19]. Research has demonstrated that manufacturing and thermal processes, including soldering and repeated bending, can alter the microstructure, reduce the mechanical strength, and compromise corrosion resistance [4, 15]. Several studies have evaluated the influence of oral environmental factors, particularly pH fluctuations and chloride ions in saliva, which are known to intensify corrosion reactions and promote localized surface breakdown [5, 17, 21]. Despite the high demand for such prostheses, especially in regions like Indonesia, research directly evaluating the corrosion resistance of stainless steel C-clasps – compared to unstressed, unbent wires of the same material – remains limited [8, 15, 17, 19]. Understanding the influence of bending is crucial for better simulation of clinical conditions.

### Objectives

This study aimed to evaluate the corrosion resistance of wrought stainless steel wire clasps at acidic (pH 5.5) and neutral (pH 7) pH levels. Specifically, this study measured the corrosion rate of stainless steel wrought wire under both pH conditions and assessed the effect of bending by comparing C-clasp specimens with straight wire on the corrosion resistance of the material.

## Methods

### Study design and setting

This experimental laboratory investigation utilized a pre–post design to evaluate the corrosion resistance of stainless steel wrought wire. The study design involved comparing straight wire again those mechanically deformed into a C-clasp under varying pH values. The fabrication of the straight wire and C-clasp specimens was conducted in Dental Laboratory, Hasanuddin University Dental Hospital, while the preparation of artificial saliva, immersion procedures, and weight measurements were performed in Microbiology Laboratory, Faculty of Medicine, Hasanuddin University. All experimental procedures were carried out between April and May 2025. Although this was an in vitro experimental study without human or animal involvement, ethical clearance was obtained from the Research Ethics Committee, Faculty of Dentistry - Dental Hospital, Hasanuddin University, Makassar, Indonesia (approval no. 082/KEPK FKG-RSGMP UH/EE/III/2025) in accordance with institutional research policy.

### Variables

The independent variable was the pH of the artificial saliva (pH 5.5 and 7). The dependent variable was the corrosion rate, which was determined by measuring the mass loss after immersion in the solution. The controlled variables included the immersion conditions (temperature, duration, volume, and solution composition) and the specimen characteristics (wire type, diameter, length, and shape).

### Specimen preparation

Stainless steel wrought wire (remanium^®^, Dentaurum, Ispringen, Germany) with a nominal diameter of 0.70 mm was used for the study. The nominal diameter was determined based on the manufacturer’s specifications. The specimens were cut to a standardized length of 50 mm (±0.05 mm). Only new, unused wires without visible defects were included. Wires exhibiting surface irregularities, plier damage, or more than one bending cycle were excluded from the study. The experimental groups consisted of straight wire at pH 5.5, C-clasp at pH 5.5, straight wire at pH 7.0, and C-clasp at pH 7.0.

All data were obtained through direct laboratory measurement. C-clasp specimens were fabricated using orthodontic pliers, and the deformation was limited to a single bending cycle. The bending radius was standardized using the same plier beak dimension for all specimens to ensure consistent curvature. The straight wire specimens were not bent. Prior to immersion, all samples were cleaned with ethanol, air dried, and weighed individually using a calibrated analytical balance with a sensitivity of 0.0001 g to record their baseline mass. Each specimen was labeled and tracked individually throughout the study period.

The artificial saliva used in this study was prepared with the following composition: sodium chloride (NaCl) 6.0 g, disodium hydrogen phosphate (Na₂HPO₄) 0.26 g, potassium thiocyanate (KSCN) 0.33 g, potassium dihydrogen phosphate (KH₂PO₄) 0.20 g, potassium chloride (KCl) 1.20 g, and sodium bicarbonate (NaHCO₃) 1.50 g, dissolved in 1000 mL distilled water. The initial pH of the solution was adjusted to 6.8. Artificial saliva solutions were prepared at pH 7 and 5.5, and the pH values were confirmed using a calibrated pH meter. For each specimen, 5 mL of artificial saliva was added to a separate sealed glass container.

### Data measurement

The specimens were fully immersed and stored in an incubator at 37°C for 672 hours (4 weeks). The immersion solution was not replaced during the experimental period. The pH of each solution was measured weekly using a calibrated digital pH meter. Measurements were performed at 7-day intervals throughout the 4-week immersion period. When deviations from the target values (pH 5.5 or 7.0) were detected, the solutions were carefully adjusted using diluted hydrochloric acid (HCl) or sodium hydroxide (NaOH) in small increments to restore the designated pH value. At each interval, the samples were removed, rinsed with distilled water, air dried, and reweighed again. The mass loss was used to calculate the corrosion rate using the standard weight-loss corrosion equation:


$$CR=\frac{K\cdot ∆W}{\rho \cdot A\cdot t}$$

where *CR* is the corrosion rate (mm/year), *K* = 8.76, Δ*W* = mass loss (g), *ρ* = material density (7.9 g/cm³, standard value for austenitic stainless steel), *A* = surface area (cm²), and *t* = time (days). The corrosion rate was calculated using the initial geometric surface area determined from the nominal dimensions of each specimen. The surface area was not recalculated weekly because the dimensional changes resulting from mass loss were minimal relative to the overall specimen geometry during the experiment.

### Bias

To reduce measurement bias, all weights were measured using the same calibrated analytical balance (resolution: 0.0001 g) under controlled conditions. The specimen length was standardized to 50 mm (±0.05 mm). The baseline masses of the 24 specimens showed limited variability (mean ± SD: 0.14135 ± 0.00338 g; range: 0.1361–0.1511 g), indicating close conformity among the samples prior to immersion. The immersion volume (5 mL), temperature (37°C), and preparation procedures were kept constant across all experimental groups. Only unused wires were included to prevent prior environmental exposure from influencing the corrosion outcomes.

### Study size

Sample size was determined using the Federer formula for completely randomized experimental designs: (*n* – 1) (*t* – 1) ≥ 15. In the present study, the experimental design consisted of two factors (pH and wire configuration), each with two levels (2 × 2 factorial design); therefore, *t* = 2. The minimum required sample size based on this calculation was *n* = 16. However, a total of 24 specimens (*n* = 6 per group) were used to provide additional replication and improve the reliability of the measurements.

### Quantitative variables

The corrosion rate was considered a continuous quantitative variable. It was calculated from the mass loss values obtained weekly over a 4-week immersion period. Group comparisons were based on the differences between the straight wire and C-clasp specimens under the two pH conditions.

### Statistical methods

Data were analyzed using the Statistical Package for the Social Sciences (SPSS). Normality was assessed using the Shapiro–Wilk test. Changes in corrosion measurements over the 4-week observation period within the same specimens were analyzed using repeated measures analysis of variance (ANOVA) for normally distributed data or the Friedman test for non-normal data. In addition, because the study followed a 2 × 2 factorial design, two-way ANOVA was used to evaluate the effects of pH, wire configuration, and their interaction on the mean corrosion rate over 4 weeks and cumulative weight loss at week 4. Statistical significance was set at *p* < 0.05.

## Results

### Participants and descriptive data

All 24 stainless steel wire specimens met the eligibility criteria and were included in the analyses. No samples were lost, damaged, or excluded during the 4-week immersion period. The specimens were evenly distributed into four groups (*n* = 6 per group): straight wire at pH 5.5, C-clasp at pH 5.5, straight wire at pH 7, and C-clasp at pH 7.

### Outcome data

**Figure 1:**
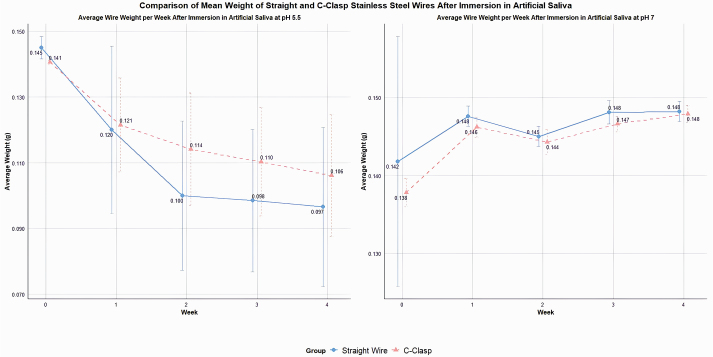
Comparison of mean weight of straight and C-clasp stainless steel wires per week after immersion in artificial saliva. The left graph shows results at pH 5.5, and the right graph shows results at pH 7.

Baseline masses demonstrated low variability (mean ± SD: 0.14135 ± 0.00338 g; variance: 1.14 × 10^–^⁵ g²; range: 0.1361–0.1511 g), indicating comparable initial conditions across the experimental groups. During immersion in saliva at pH 5.5, both the straight and C-clasp specimens exhibited progressive weight loss, with the straight wire exhibiting a greater decrease. The largest mass loss occurred during the first week of immersion, followed by a gradual decline in the mass loss rate in subsequent weeks. In contrast, specimens immersed in artificial saliva at pH 7 demonstrated minimal changes, with values fluctuating slightly over the weeks. The weight change patterns are illustrated in Figure 1.

**Figure 2:**
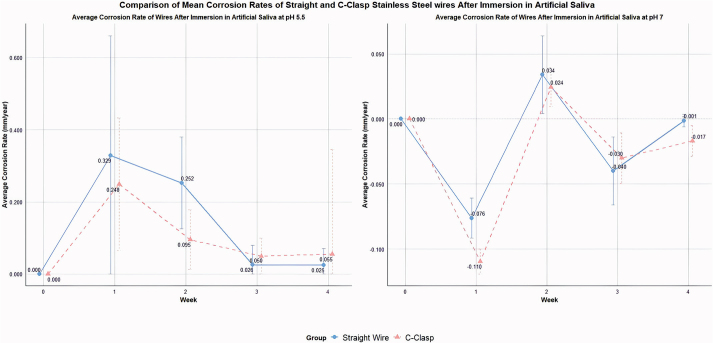
Comparison of mean corrosion rates of straight and C-clasp stainless steel after immersion in artificial saliva. The left graph shows results at pH 5.5, and the right graph shows results at pH 7. Corrosion rates are expressed in mm/year (annualized values derived from weekly measurements).

The corrosion rates were calculated individually for each specimen based on the mass change measured at each time interval. The highest corrosion rates were observed during week 1, particularly for straight wires immersed in pH 5.5 solution. The rates declined steadily over the subsequent weeks. At pH 7, the corrosion rates were substantially lower and fluctuated around zero, with occasional negative values indicative of minor measurement variations rather than true metal gain. The weekly corrosion rates for each group are presented in Table 1, and the trends are shown graphically in Figure 2.

**Table 1:** Mean corrosion rate of stainless steel wires per week after immersion in artificial saliva at pH 5.5 and pH 7.

Wire form	Weeks				
	0	1	2	3	4
**pH 5.5**					
Straight wire	0	0.329 ± 0.332	0.252 ± 0.127	0.026 ± 0.053	0.025 ± 0.046
C clasp	0	0.248 ± 0.184	0.095 ± 0.083	0.050 ± 0.050	0.055 ± 0.290
**pH 7**					
Straight wire	0	-0.076 ± 0.002	0.034 ± 0.030	-0.040 ± 0.026	-0.001 ± 0.005
C clasp	0	-0.110 ± 0.010	0.024 ± 0.015	-0.030 ± 0.019	-0.017 ± 0.012

Notes: Values are presented as mean ± standard deviation (SD); *n* = 6 specimens per group.

### Main results

#### Weight change over time

Two-way ANOVA (Table 2) revealed that pH significantly affected the cumulative weight loss at week 4. Specimens immersed in artificial saliva at pH 5.5 exhibited greater weight loss than those immersed at pH 7.0. In contrast, the wire configuration and the interaction between pH and wire configuration had no significant effect. Levene’s test indicated unequal variances; therefore, these findings were interpreted cautiously.

**Table 2:** Two-way ANOVA results for cumulative weight loss at week 4.

Source	Comparison / Effect	Mean ± SD	*P*	Significance
pH	pH 5.5	29.037 ± 15.006	< 0.001	Significant
	pH 7.0	-5.964 ± 1.618		
Wire configuration	Straight wire	14.477 ± 22.820	0.189	Not significant
	C-clasp	8.596 ± 18.878		
pH × wire configuration	Interaction effect		0.491	Not significant

Note: Values are presented as mean ± standard deviation (SD). The dependent variable was the cumulative weight loss at week 4. Levene’s test indicated unequal variances, *F*(3,20) = 9.464, *p* < 0.001; therefore, the results were interpreted cautiously.

#### Corrosion rate

Two-way ANOVA (Table 3) revealed a similar pattern for the mean corrosion rate over 4 weeks. Thus, pH had a significant effect, with specimens immersed at pH 5.5 exhibiting higher corrosion rates than those immersed at pH 7.0. The wire configuration and the interaction between pH and wire configuration had no significant effects.

**Table 3:** Two-way ANOVA results for mean corrosion rate over 4 weeks.

Source	Comparison / Effect	Mean ± SD	*P*	Significance
pH	pH 5.5	0.510 ± 0.254	< 0.001	Significant
	pH 7.0	-0.101 ± 0.024		
Wire configuration	Straight wire	0.266 ± 0.413	0.096	Not significant
	C-clasp	0.144 ± 0.301		
pH × wire configuration	Interaction effect		0.238	Not significant

Note: Values are presented as mean ± standard deviation (SD). The dependent variable was the mean corrosion rate for 4 weeks.

## Discussion

This study demonstrated that environmental pH plays a more critical role in the corrosion behavior than the wire configuration. These findings were supported by the observed differences between acidic and neutral environments. Thus, specimens immersed in artificial saliva at pH 5.5 exhibited greater mass loss than those at pH 7, indicating increased corrosion under acidic conditions. This finding aligns with the known susceptibility of stainless steel to accelerated corrosion in acidic environments, which destabilizes its protective chromium oxide passive layer [22, 23]. In contrast, the wire configuration had no significant effect on either the corrosion rate or cumulative weight loss, and no significant interaction was observed between pH and wire configuration.

Previous studies have reported that bending can introduce localized surface stresses or microstructural changes that may affect the integrity of the passive oxide layer and thereby influence corrosion behavior [11, 22]. Nevertheless, such effects are not always pronounced, particularly under neutral conditions, where stainless steel generally maintains its corrosion resistance. Specifically, no significant or progressively worsening corrosion associated with bending was observed in this study. However, given the limited sample size, the absence of an observed effect of bending on corrosion should be interpreted with caution.

Under neutral conditions (pH 7), several specimens showed small fluctuations in weight during the 4-week immersion period, including slight increases in mass at certain time points. Similar observations have been reported in corrosion studies of stainless steel under neutral conditions, which are possibly related to surface reactions during immersion [11, 13]. Under neutral conditions, stainless steel typically maintains a stable chromium-rich passive oxide layer, which may undergo cycles of breakdown and repassivation [11]. The formation or thickening of surface oxide films and the adsorption of ions from the surrounding solution may temporarily increase the specimen weight despite ongoing corrosion reactions. In addition, the corrosion rate and total mass loss are not always directly proportional to each other. Previous studies have suggested that localized corrosion processes, such as pitting, can occur without substantial overall weight loss [14]. However, because this study evaluated corrosion primarily through mass change measurements and did not include surface characterization, the occurrence of such localized corrosion could not be confirmed. Further investigations involving ion release analysis and surface examination techniques are required to clarify the mechanisms responsible for these fluctuations.

From a clinical perspective, the metal clasp components of removable partial dentures are continuously exposed to complex oral conditions. Clinical studies have reported increased plaque accumulation and biological changes around abutment teeth, reflecting the combined influence of mechanical, chemical, and biological factors [24]. Similarly, intraoral metallic appliances, including prosthodontic and orthodontic devices, are subject to biofilm accumulation and corrosion-related surface changes in the oral environment, which may contribute to material degradation over time [18, 25]. These clinical conditions may create localized environments with altered pH, thereby supporting the relevance of the present findings in acidic conditions.

However, several limitations should be considered when interpreting these findings. The present study was conducted under controlled in vitro conditions using static artificial saliva without salivary flow or temperature fluctuations, which does not fully represent a dynamic oral cavity environment. Additionally, the absence of microbial biofilms may have influenced the corrosion behavior, as microbial metabolic activity and pH changes can affect the electrochemical reactions on metal surfaces. Manual bending of the wire may also introduce minor variations in the deformation although the same operator and tools were used to maintain consistency. The immersion period was limited to 4 weeks, allowing for the observation of early corrosion behavior but not the long-term degradation trends. Future studies incorporating dynamic saliva flow, temperature cycling, mechanical loading, biofilm models, and longer immersion periods are required to better simulate clinical conditions and to validate these findings.

Taken together, the findings of this study provide initial evidence regarding the corrosion behavior of stainless steel wrought wires used for clasp fabrication in removable partial dentures. The observed corrosion pattern was consistent with previously reported mechanisms, and the significant effect of pH was expected, with acidic conditions accelerating material degradation compared to normal environments. In contrast, the absence of a significant difference between the straight and C-clasp configurations was unexpected, as mechanical deformation was hypothesized to influence corrosion susceptibility. It is possible that the limited sample size reduced the statistical power to detect the subtle effects of wire bending. Therefore, these results highlight the need for further investigations with larger sample sizes and complementary analytical techniques to clarify whether the wire configuration contributes to distinct corrosion patterns.

## Conclusion

Acidic environments significantly increased the corrosion rate and mass loss of stainless steel wrought wire, whereas neutral conditions promoted its surface stability, with no significant differences in the corrosion behavior between the straight and C-clasp wires.

## Other information

### Data availability statement

The data supporting this study are available upon reasonable request to the corresponding author.
